# In Vitro Starch Digestibility and Glycaemic Index of Fried Dough and Batter Enriched with Wheat and Oat Bran

**DOI:** 10.3390/foods9101374

**Published:** 2020-09-27

**Authors:** Oluwatoyin O. Onipe, Daniso Beswa, Afam I. O. Jideani

**Affiliations:** 1 Department of Food Science and Technology, School of Agriculture, University of Venda, Thohoyandou 0950, South Africa; toyin.onipe@gmail.com; 2 Department of Biotechnology and Food Technology, Faculty of Science, University of Johannesburg, Doornfontein 2028, South Africa; beswad@uj.ac.za

**Keywords:** in vitro assay, glycaemic index, bran, digestible starch, fried dough, *magwinya*

## Abstract

A deep-fried dough/batter food (*magwinya*) consumed across different age groups and social strata in South Africa was investigated in this study for digestibility and estimated glycaemic index (eGI). In this research, we investigated the influence of bran type—wheat bran (WB) and oat bran (OB), and concentration (0–20% *w/w*) on the starch digestibility and eGI of *magwinya*. Rapidly available glucose (RAG) of control fried dough (60.31 g/100 g) was 33% less than fried batter (90.07 g/100 g). There was a significant reduction in RAG and an increase in slowly available (SAG) and unavailable glucose (UG) content of the fried products with OB and WB addition. The highest SAG content was observed in WB fried dough. Control fried batter had the highest eGI value (80.02) and control fried dough had medium eGI value (58.11). WB fried dough, fried batter, and OB fried dough were categorised as medium GI foods at eGI range of 56.46–58.39, 65.93–68.84 and 56.34–57.27, respectively. The eGI values of OB fried batter ranged from 73.57 to 80.03 and were thus classified as high GI foods. UG showed significant correlation with eGI (r = −0.892, −0.973, *p* < 0.01) and fat content (r = −0.590, −0.661, *p* < 0.01) for WB and OB fried products. These results reveal that ingredient modification through bran enrichment is effective for the regulation of starch digestion and reduction of eGI of deep-fried dough/batter foods.

## 1. Introduction

Starch is the major carbohydrate found in food commodities such as maize, wheat, potato and rice [1]. It is a good source of energy for humans, however, its excessive consumption is of health concern, as it is a predisposing factor to other metabolic-related diseases like obesity, and diabetes—A projected leading cause of death by the year 2030 according to the World Health Organisation [2,3]. Moreover, the ability to predict and regulate postprandial glucose absorption of starchy food is vital to the global epidemic called diabesity [4,5]. Starch digestion (SD) is characterised by the rate and the duration of postprandial glycaemic response. Starch can be undigested, rapidly or slowly digested. Starch granule characteristics, state, size, processing methods and presence of other ingredients all influence SD [6].

Understanding the relationship between the physicochemical and physiological properties of food is a vital way to explain the value of carbohydrate in human nutrition. The glycaemic glucose fraction of food, excluding lactose, is a summation of glucose in its glycaemic carbohydrate fraction [7]. The glucose content of a carbohydrate-rich food is categorised as either rapidly (RAG) or slowly available (SAG) to signify its potential rate of release and absorption or non-absorption for unavailable glucose (UG) [6]. The rate of postprandial glucose release can be described by its in vitro RAG and SAG values which are the main determinants of the glycaemic index of cereal-based foods [6,7]. Glycaemic index (GI) classifies carbohydrate foods based on how they influence postprandial plasma glucose response [8]. GI is expressed as the percentage increase in the glucose area under the curve of a test food against a standard food such as glucose or white bread [7,9]. GI is usually measured in vivo, however, because of the time and resources required, in vitro methods have been adopted over the years as a relevant nutromic analysis tool to measure the rate of hydrolysis and assess the glycaemic index of foods [9,10].

Factors that affect starch digestibility include starch characteristics, enzyme inhibitors, amylose/amylopectin ratio, particle size of starch granule, processing method, starch gelatinisation, retrogradation and presence of other ingredients such as lipids, proteins and fibre [4,6,11,12]. Previous studies present persuasive evidence that certain dietary fibres noticeably reduced GI of foods, although the magnitude of GI reduction is dependent on the food type and processing involved [13,14]. Wheat (WB) and oat bran (OB) are cheap, easily accessible, fibre-rich food additives which, when incorporated into starchy foods, may reduce their GI. The in vitro hypoglycaemic effects of WB and OB include an increase in slowly digestible starch (SDS) and resistant starch (RS) contents of foods [15,16,17,18]. The resistance of dietary fibres to amylolytic enzymes in the small intestine results in their transformation to short-chain fatty acids in the colon. These processes result in physiological benefits such as blood glucose regulation, laxative and prebiotic properties [14,19].

*Magwinya* is a deep-fried product made from wheat flour, sugar, salt and water. It can be categorised as fried dough or batter based on the preferences of the consumers [20]. It is popularly consumed by people of various age groups in sub-Saharan Africa [20] and can be categorised as a high-carbohydrate and food because of its major ingredient–refined wheat flour. Owing to its prevalent consumption as the main meal amongst low-income population [20], it is essential to improve its nutritional quality. To the best of our knowledge, in vitro starch digestion and GI of *magwinya* has not been reported. Therefore, the objectives of this paper are: (i) to quantify available glucose and digestible starch fractions of *magwinya* via in vitro assay; (ii) to assess the effect of bran concentration and initial moisture content on available glucose and digestible starch content of *magwinya*; and (iii) to calculate the estimated glycaemic index of *magwinya.*

## 2. Materials and Methods 

### 2.1. Materials

Wheat flour (carbohydrates—71%, protein—10%, moisture—12%, fibre—4%, fat—1%) wheat bran (carbohydrates—22%, protein—16%, moisture—11%, fibre—43%, fat—5%), oat bran (carbohydrate—50%, protein—13%, moisture—7% fibre—20%, fat—5%,), sucrose (Selati, RCL Foods), salt, instant yeast (Anchor Yeast), sunflower oil (Spar) sourced locally in South Africa were used for the production of fried products. Wheat and oat bran were milled to 200 µm particle size using an ultra-centrifugal mill (Retsch ZM 200, Haan Germany) and were stored at –20 °C until used for *magwinya* production. Guar gum (G4129), amyloglucosidase (A7095–260 U/mL), pepsin (P7000–250 U/mL), invertase (I4504–300 U/mg) and pancreatin (P7545–8 x USP specification), sulfuric acid (25810-5), glucose oxidase peroxidase kit (GAGO 20) for in-vitro digestion assays were sourced from Sigma–Aldrich, (St Louis, MO, USA). Potassium hydroxide, acetic acid, hydrochloric acid and other chemicals used were of analytical grade.

### 2.2. Production of Fried Products (Magwinya)

Fried products were processed using the method of Onipe et al. [21]. Wheat flour was partially substituted with 1, 5, 8, 10, 15 and 20% (*w/w*), respectively, of wheat and oat bran. All dry ingredients were weighed as follows: composite flour (100 g), sugar (15 g), salt (1 g), yeast (1 g), into a mixing bowl at ambient temperature (24 °C). Lukewarm water (65 mL and 100 mL) was added gradually until all ingredients were homogenously mixed to form a dough and batter, respectively. The dough was kneaded for 10 min in a mixer (Russell Hobbs RHSB237, Failsworth, UK) and cut into a 50-g mass and formed into a ball. The dough and batter were proofed at 30 °C in a T7 bread proofer (with temperature control limit of 46 °C and 95% humidity) and fried at 180 °C for 5 min in a deep fryer (Russell Hobbs RDF300, Failsworth, UK) with built-in automatic temperature and time control system. Oil was pre-heated for 1 h before frying.

### 2.3. Sample Preparation for Starch Digestion

**Figure 1:**
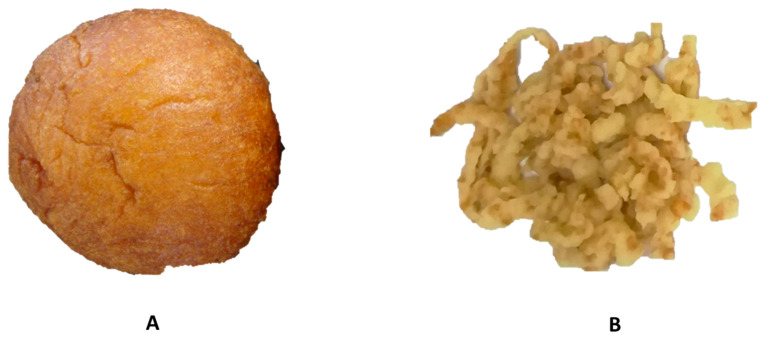
Whole (**A**) and minced (**B**) fried batter.

Various sample crushing methods have been reported such as milling/grinding, mincing and chewing. Mincing was selected because it gave consistent results close to chewing in cereal foods [22]. To keep analysis as close as possible to real human experience, samples were analysed fresh as opposed to some analysis where samples were dried before digestion. For each sample, one whole freshly fried *magwinya* was minced (Figure 1) using an electronic meat grinder (Brabantia BBEK1092V, Brabant, Netherlands) equipped with a 0.9-cm diameter hole [23].

### 2.4. Starch Digestibility Protocol

In vitro starch digestibility of *magwinya* was determined using the method described by Englyst et al. [23,24] and Cortado et al. [1] with modifications in the following three steps:

Step 1: Incubation of fried products with pepsin for proteolysis

Approximately 1.5 g of minced sample was transferred to 50 mL centrifuge tubes. Then, 5 mL of 50% saturated benzoic acid solution and 10 mL of pepsin guar-gum solution (5 g each per litre of 0.05 M HCl) was added. Guar gum was added to standardise the viscosity of the solution. To initiate proteolysis, each tube was covered, vortex-mixed, and transferred to a water bath for 30 min at 37 °C. To each tube, 5 mL sodium acetate buffer solution (0.5 M) at pH 5.2 and 15 glass beads of 5 mm diameter were added, shaken gently and equilibrated for 3 min in the water bath.

Step 2: Starch hydrolysis of fried products

Fresh enzyme cocktail was made from amyloglucosidase (260 U/mL), invertase (300 U/mg) and pancreatin (8 × USP) at 4:6:18 ratio per 100 mL enzyme mixture. Five millilitres of enzyme cocktail was added to each tube and agitated at 137 rpm in a shaking water bath set at 37 °C. Each tube was timed and removed from the water bath at exactly 20 and 120 min after the addition of the enzyme cocktail. Approximately 0.2 mL of the digest was pipetted into 4 mL absolute ethanol and vortex-mixed to end enzymatic hydrolysis. These were G20 and G120 fractions (glucose concentration at 20 min and 120 min).

Step 3: Digestion for hydrolysis of starch to glucose 

The tubes were covered, mixed vigorously on a vortex mixer, and cooled for 5 min in an ice water bath. Subsequently, 7 M KOH solution (10 mL) was added and mixed. The tubes were agitated horizontally for 30 min in an ice water bath. A 0.2-mL portion of the content was pipetted into 1 mL acetic acid 1 M solution after which 40 µl of amyloglucosidase solution was added. The tubes were vortex mixed and transferred to a water bath for 30 min at 70 °C. The tubes were cooled for 15 min in an ice bath and subsequently allowed to reach ambient temperature before adding 12 mL of absolute ethanol. This tube corresponded to the TG (total glucose) portion. Glucose oxidase and peroxidase assay kit were used to estimate glucose concentration of G20, G120, and TG portions using glucose oxidase and peroxidase assay kit GAGO-20 (Sigma–Aldrich, St Louis, MO). The RAG, SAG and UG fractions were estimated using equations 1–3 as described by Contardo et al. [1].

(1) $$\mathrm{Rapidly}\;\mathrm{available}\;\mathrm{glucose}\;\left(g/100\;g\right){\;=\;G}_{20}/\mathrm{TG}\times \;100$$

(2) $$\;\mathrm{Slowly}\;\mathrm{available}\;\mathrm{glucose}\;\left(g/100\;g\right)\;=\;\left(G_{120}{\;-\;G}_{20}\right)/\mathrm{TG}\;\times \;100\;$$

(3) $$\;\mathrm{Unavailable}\;\mathrm{glucose}\;\left(g/100\;g\right)\;=\;\left({\mathrm{TG}\;-\;G}_{120}\right)/\mathrm{TG}\;\times \;100\;$$

### 2.5. Glycaemic Index Estimation of Fried Products

The kinetics of starch hydrolysis was described by the non-linear first-order Equation (4) established by Goñi et al. [25]. The estimated glycaemic index of *magwinya* was calculated from Equation (5).

(4) $${C\;=\;C}_{\infty }\;\left({1-\;e}^{-\mathrm{kt}}\right)$$

(5) Estimated glycaemic index = 39.71 + 0.548 × Hydrolysis index

The hydrolysis index (HI) was obtained from a division of area under the hydrolysis curve (AUC) of *magwinya* sample and AUC of the reference sample (white bread) as presented in the Equations (6) and (7) [25,26].

(6) $$\mathrm{Hydrolysis}\;\mathrm{index}\;=\;\frac{{\mathrm{Area}\;\mathrm{Under}\;\mathrm{Curve}}_{\mathrm{magwinya}}}{{\mathrm{Area}\;\mathrm{Under}\;\mathrm{Curve}}_{\mathrm{white}\;\mathrm{bread}}}\;\;$$

(7) $${\mathrm{Area}\;\mathrm{under}\;\mathrm{curve}\;=\;C}_{\infty }\left(t_{f}{-\;t}_{0}\right)-\left(C_{\infty }/k\right)\;\left[{1-\;e}^{-k\;\left(t_{f}{-\;t}_{0}\right)}\right]$$

where C_∞_ is the equilibrium concentration at the final time (120 min) and k is the kinetic constant. t_f_ is final time t_0_ is the initial time, C is the starch hydrolysed at a chosen time t.

### 2.6. Statistical Analysis

Results were presented as the mean of triplicate determinations. Analysis of variance was carried out to determine the effect of bran concentration on glucose fractions. Means were separated using Duncan’s multiple range test where the effect of the independent variable was significant at *P <* 0.05. Multivariate linear regression was carried out to determine the test of between-subject effect, that is, linear and interaction effect of bran addition, initial moisture content and bran type on dependent variables. The association between independent and dependent variables was tested using Pearson’s correlation. All statistical tests were performed using SPSS software (SPSS statistics version 26, IBM Co., Armonk, NY, USA).

## 3. Results and Discussion

### 3.1. Rapidly and Slowly Available Glucose Content of Fried Products

The glucose fractions of *magwinya* have their source linked to starch and sugar components of *magwinya*. The RAG and content of starchy food are significant determinants of its glycaemic response [27]. The magnitude of the in vivo GI of most carbohydrate-rich foods is almost certainly determined by the RAG content [23,26]. This is backed up in literature with a strong correlation of GI and RAG content for several starchy foods [27]. RAG values of WB of fried dough ranged from 47.11 to 60.31 g/100 g while that fried batter ranged from 72.33 to 90.07 g/100 g (Table 1) and decreased with an increase in WB content for fried dough and batter. These values were close to the RAG values of the deep-fried dough matrix reported by Contardo et al. [1]. RAG content was highest in control samples followed by a significant decrease was noted up to 10% WB substitution, followed by an increase thereafter in WB fried dough and batter. As initial moisture content increased, RAG values also increased (r = 0.931, *p <* 0.01) implying that the consumption of WB fried batter will likely cause the rapid release of glucose in the bloodstream. The SAG content of WB fried dough ranged from 2.62 to 12.56 g/100 g and 6.88 to 18.76 g/100 g in fried batter (Table 1). SAG was lower in WB fried dough compared to the fried batter. The SAG content in both sets of samples was below 20 g/100 g and increased significantly with increments of WB up to 10%. 

The RAG of OB fried dough ranged from 41.47 to 60.31 g/100 g while that of fried batter ranged from 71.84 to 90.07 g/100 g (Table 1). A significant reduction was observed up to 10% OB in fried batter. In OB fried dough, there was a significant reduction of RAG values with an increase in bran concentration. Initial moisture content and bran concentration were positively correlated to RAG content of OB (0.942) and WB *magwinya* (0.931) at *p <* 0.01. Higher RAG values in fried batter may be indicative of a higher degree of gelatinisation of fried batter compared to fried dough. Gelatinised starch is easily hydrolysed because of the loss of amylopectin crystallinity which makes the granules porous and accessible to digestive enzymes [1,13]. Control samples had the highest RAG values (fried batter—90.07 g/100 g, fried dough—60.31 g/100 g); which implies that the inclusion of OB reduced RAG values in both fried products. Human/animal subjects were not used for the study. However, this result implies that the incorporation of OB in *magwinya* formulation may delay the release of glucose in the bloodstream within 20 min of digestion. The SAG content of OB fried dough ranged from 2.62 to 10.18 g/100 g and 6.88 to 14.07 g/100 g in fried batter (Table 1). A significant increase in SAG content was noted at 5% OB in the fried dough and 1% OB in fried batter, respectively. Pearson’s correlation showed that the increase in initial moisture content increased SAG of WB (r = 0.596) and OB *magwinya* (r = 0.557) at *p <* 0.01.

### 3.2. Unavailable Glucose Content of Fried Products

The UG fraction corresponds to the amount of glucose not released within 120 min of starch digestion and it is mainly from resistant starch content of the food [1]. The UG values of WB fried dough and batter ranged from 35.27 to 45.44 g/100g and 3.05 to 9.57 g/100 g, respectively. These results showed that UG values were higher in the fried dough than in fried batter. In WB fried dough, a significant increase in UG was observed from 8% WB while that of fried batter showed an increase from 1% WB. The UG values of OB fried dough and batter ranged from 37.07 to 54.74 g/100 g and 3.05 to 16.68 g/100 g, respectively. The UG values in OB *magwinya* are higher than those for WB *magwinya.* This may be associated with the difference in fibre solubility of WB and OB. The rate of UG increase is like those observed in WB fried dough and batter. An increase in UG in fried batter may be attributed to the interaction of WB fibres with water at a molecular level [28]; thus, reducing the water available for starch gelatinisation, and as such, digestive enzymes could not hydrolyse the starch at final digestion time. The mean UG values reported in this study were close to those of Contardo et al. [1] where UG of dough matrix fried under atmospheric conditions was 46 g/100 g. A negative correlation was established for initial moisture content and UG of WB *magwinya* (r = −0.985) and OB *magwinya* (r = −0.935) at *p <* 0.01. The UG values of OB fried batter increased significantly (*p <* 0.05) at higher substitution levels of OB.

High UG values in fried dough samples may also be accounted for by incomplete gelatinisation as a result of competition for water by fibre, salt, and sugar molecules causing less water for starch granules to attain full gelatinisation upon heating [1,6]. Multivariate linear regression revealed that linear and interaction effect of the independent variables (bran type, initial moisture content and bran concentration) all showed significance (*p <* 0.05) on RAG, SAG and UG contents of *magwinya* except for interaction effect of bran type and initial moisture content which showed no significance (*p* > 0.05) on SAG

### 3.3. Estimated Glycaemic Index of Fried Products

#### 3.3.1. Estimated Glycaemic Index of Fried Dough

The eGI values of WB fried dough were in the range of 56.46–58.39 and were significantly lower than control (58.11) except WB1 (Table 2). The eGI of OB fried dough ranged from 56.34 to 57.27 and was significantly lower than control (58.11), but no statistical difference was observed among the samples. This implies that *magwinya* with low initial moisture content (fried dough) are medium GI food. This reduction in eGI of fried dough samples may be attributed to the combined effect of dietary fibre and limited water in the food matrix, which would have caused incomplete starch gelatinisation, and of a consequence resistance to amylolytic enzymes in the digestion time frame [3]. In a food matrix such as *magwinya,* which has other components such as sugar (sucrose), salt, protein (gluten) and fibre, coupled with low initial water in the dough, these components compete with the starch granules for water. For complete gelatinisation to occur, heat and water are two crucial factors viz: water must enter the crystalline region (amylopectin) where swelling occurs (upon heating) and the cell ruptures, causing the amylose to leach out. It is easier for digestive enzymes to hydrolyse the exposed amylopectin and amylose into glucose monomers for absorption in the body. When there is incomplete gelatinisation, the foregoing does not occur, and this reduces the GI of the food [29].

#### 3.3.2. Estimated Glycaemic Index of Fried Batter

Fried batter exhibited high eGI values viz: 65.93–80.62 in WB samples and 73.75–80.62 in OB samples (Table 2). The linear effect of initial moisture content and its interaction effect with other independent variables showed significance (*p <* 0.05) on eGI of OB and WB *magwinya*. There was a significant reduction in eGI of *magwinya* with increments to OB and WB concentrations in *magwinya* formulation. In the control sample, eGI of 80.62 revealed fried batter to be a high GI food, while that of fried dough at 58.11 can be classified as intermediate GI food.

Carbohydrate-rich foods can either be labelled low GI (<55), medium (55–70) and/or high GI (> 80) [30]. The eGI of OB fried batter samples were all above 70 indicating that fried batter samples, despite OB addition are high GI foods. WB fried batter samples, on the other hand, were in the 65–68 range and were significantly (*p <* 0.05) lower than their OB counterpart. In the glycaemic index table collated by Foster-Powell et al. [31], GI of doughnut from an in vivo study was reported as 76 and 108 when glucose and white bread were used as the reference food, respectively. Fried dough samples exhibited lower GI than fried batter samples.

The GI-lowering effect of bran types used in this study can be linked to their dietary fibre content which is composed mostly of non-starch polysaccharides such as β glucans (soluble fibre) in OB and arabinoxylan in WB [19,32]. The soluble fibre of OB acts through alteration of the food microstructure via restriction of starch gelatinisation because of its high-water absorption capacity which in turn limits water available to starch granules in the food matrix [3,15]. Wheat bran is composed of up to 58% insoluble dietary fibre which increases faecal bulk, reduces transit time in the small intestine and is resistant to enzymatic hydrolysis due to its heterogeneous structure [19]. This resistance to digestive enzymes may have contributed to lowering the eGI of our fried products. The eGI of WB samples was lower than OB samples for both fried batter and dough; thus, deducing that WB is a better choice at lowering the GI of *magwinya* made from either dough/or batter. Despite the similarity between both types of products, their eGI shows high disparity which was affected by the difference in ingredient (bran type used) and initial moisture content of the products which resulted in different degrees of starch gelatinisation and rates of carbohydrate digestion and their respective eGI values.

### 3.4. Relationship Between Estimated Glycaemic Index, and Other Variables in the Study

The relationship between GI, glucose, and total oil content of *magwinya* was estimated using Pearson’s correlation to observe and report linear trends amongst these properties. The fat content of the fried products in this study was reported in a recent publication [21].

#### 3.4.1. Wheat Bran Fried Products

The relationship between glucose (RAG, SAG, UG), fat content and eGI of WB and OB *magwinya* is presented in Table 3. There was a positive correlation between eGI and RAG (r = 0.916, *p <* 0.01) and SAG (r = 0.338, *p <* 0.01) of WB *magwinya*, implying that increase in SAG and RAG may have led to an increase in eGI. The RAG of foods has been established as a potential predictor of in vitro glycaemic response of starchy foods [27]. Initial moisture content (r = 0.931, *p <* 0.01) correlated positively to RAG (r = 0.931, *p <* 0.01) and SAG (r = 0.596, *p <* 0.05). This supports the effect of high water in fried batter samples that caused complete gelatinisation for fried batter. For complete gelatinisation during heat treatment, water is essential to hydrate starch molecules and cause a disruption of the crystalline structure of amylopectin. When there is not enough water, gelatinisation becomes incomplete which affects starch digestion [33]. The WB concentration correlated negatively to RAG (r = −0.310, *p <* 0.05) and positively to SAG (r = 0.488, *p <* 0.01). Fat content correlated positively to RAG and SAG of WB *magwinya* and negatively to UG (r = −0.590, *p <* 0.01). Individually in fried dough, a negative correlation was established between FC and UG (r = −0.749, *p <* 0.01), while no significant correlation existed in WB fried batter. A positive correlation between fat and eGI (r = 0.495, *p <* 0.01) showed that an increase in fat content may increase eGI. This may be accounted for by fried batter samples which had higher eGI values. A possible explanation could be the enzyme used which catalysed fried batter samples thus increasing the eGI content. The fat content of fried products was inversely related to UG and directly proportional to eGI–indicative of amylose–lipid complex formation in the fried dough and starch gelatinisation in fried batter products [34].

#### 3.4.2. Oat bran Fried Products

Correlation among independent variables, available glucose (RAG, SAG, UG), and eGI of OB *magwinya* is presented in Table 3. Like WB products, RAG and SAG contributed strongly to eGI of OB *magwinya* as indicated by positive correlations (r = 0.962, 0.518, *P <* 0.01). Initial moisture content positively correlated to RAG, SAG and eGI of OB *magwinya* as shown (Table 3), and negatively correlated to UG (r = −0.963, *p <* 0.01). In a low-moisture system like fried dough in this study, an increase in UG may be as a result of incomplete starch gelatinisation. Abundant water and heat are two important factors that aid starch gelatinisation and in turn the extent of starch digestion during hydrolysis. Exposure of starch molecules to heat in excess water leads to disruption of the crystalline structure thereby linking the hydroxyl group of starch and water molecules via hydrogen bonding [12]. This, in turn, solubilises the starch granule, leading to increased swelling and subsequent leaching of amylose which is easily accessible to amylolytic enzymes during digestion [6]. Correlation of fat content to glucose and starch contents revealed similar results as WB *magwinya*. There is a dearth of research on starch digestion of deep-fried dough/batter foods, hence the comparison of the results in this study with other deep-fried starchy foods. Fat content negatively correlated to UG (r = −0.661, *p <* 0.01). Opposing results on the effect of deep-frying on RS content of potato products have been reported. Deep-frying significantly increased RS because deep-frying increases fat available for amylose–lipid complex formation in the food [34,35]. Starch–lipid complexes in food matrices are characterised by reduction of starch solubility, swelling power, gelatinisation and retrogradation which in turn delays enzymic digestion [11]. Conversely, Yadav [36] observed that deep-frying had a prominent effect on RS reduction. In this study, however, the percentage of fat content was compared to the starch contents and an inverse relationship existed between RS and fat content for OB and WB fried products. The negative correlation can be linked to starch gelatinisation which was more pronounced in batter samples (having higher fat content) because of higher initial water content. The inclusion of bran in *magwinya* formulation also contributed to fat reduction and consequential increase in RS [21].

## 4. Conclusions

Incorporation of WB and OB in *magwinya* fried products caused a reduction in RAG values as well as an increase in SAG and UG values. This points to a delay in glucose release due to the presence of bran fibres during enzymatic hydrolysis. OB and WB had a similar effect on the reduction of RAG of fried dough while WB had improved an effect in fried batter products. There was a reduction of eGI of all samples with addition of WB and OB. This reduction effect on the eGI of *magwinya* can be attributed to the dietary fibre constituents of OB and WB. Overall, this study shows how bran enrichment can be used to control the glucose contents and reduce eGI of *magwinya*, thereby improving its nutritional properties. The outcomes of this study can serve as baseline information for *magwinya* (or fried food) processors, nutritionists, and consumers. In vitro studies only cater to an estimation of starch digestion and glycaemic index. To obtain *magwinya* GI, in vivo study using human or animal subjects is recommended. Extraction and purification of bran dietary fibres and evaluation of their constituents on their molecular mechanism in starch digestion are also suggested for future studies. The results of this study indicated that WB and OB are suitable additives for the reduction of RAG and eGI of *magwinya.*

## Figures and Tables

**Table 1:** Glucose content (g/100 g) of fried products enriched with wheat and oat bran.

Bran Concentration (g)	Fried Dough			Fried Batter		
	RAG	SAG	UG	RAG	SAG	UG
Control	60.31 ^e^ ± 1.51	2.62 ^a^ ± 0.03	37.07 ^ab^ ± 0.24	90.07 ^e^ ± 1.80	6.88 ^a^ ± 0.78	3.05 ^a^ ± 0.61
WB1	56.81 ^d^ ± 0.12	4.69 ^b^ ± 0.20	38.50 ^b^ ± 1.08	83.17 ^d^ ± 1.13	9.45 ^b^ ± 0.43	7.39 ^c^ ± 0.30
WB5	53.67 ^c^ ± 1.31	11.06 ^de^ ± 0.42	35.27 ^a^ ± 0.11	80.14 ^c^ ± 0.25	10.93 ^b^ ± 0.62	8.93 ^d^ ± 0.36
WB8	48.10 ^a^ ± 0.20	10.45 ^d^ ± 0.05	41.46 ^c^ ± 0.15	76.09 ^b^ ± 0.05	18.76 ^d^ ± 0.05	5.15 ^b^ ± 0.01
WB10	49.18 ^ab^ ± 0.85	12.56 ^e^ ± 0.12	38.26 ^b^ ± 0.27	73.38 ^a^ ± 0.58	17.99 ^d^ ± 0.76	8.63 ^d^ ± 0.18
WB15	51.12 ^b^ ± 0.33	5.67 ^bc^ ± 0.17	43.21 ^c^ ± 1.63	78.58 ^c^ ± 0.18	14.77 ^c^ ± 0.68	6.65 ^c^ ± 0.51
WB20	47.11 ^a^ ± 0.25	7.45 ^c^ ± 0.50	45.44 ^d^ ± 1.25	72.33 ^a^ ± 1.78	18.11 ^d^ ± 0.85	9.57 ^d^ ± 0.07
Control	60.31 ^c^ ± 1.51	2.62 ^a^ ± 0.03	37.07 ^a^ ± 0.24	90.07 ^d^ ± 1.80	6.88 ^ab^ ± 0.78	3.05 ^a^ ± 0.61
OB1	45.70 ^b^ ± 1.77	2.48 ^a^ ± 0.48	51.82 ^c^ ± 1.30	82.25 ^c^ ± 0.28	14.07 ^c^ ± 0.78	3.68 ^a^ ± 0.59
OB5	44.27 ^b^ ± 0.11	4.35 ^ab^ ± 0.21	51.38 ^c^ ± 0.11	76.46 ^ab^ ± 0.72	9.72 ^c^ ± 0.59	13.82 ^c^ ± 0.13
OB8	43.71 ^ab^ ± 1.03	8.45 ^c^ ± 0.98	47.84 ^b^ ± 0.21	75.41 ^ab^ ± 0.91	13.49 ^c^ ± 0.76	11.10 ^b^ ± 0.15
OB10	41.47 ^a^ ± 0.25	3.79 ^a^ ± 0.10	54.74 ^d^ ± 1.15	71.84 ^a^ ± 0.75	11.30 ^bc^ ± 0.71	16.86 ^d^ ± 0.96
OB15	43.25 ^ab^ ± 0.40	10.18 ^c^ ± 0.41	46.57 ^b^ ± 0.21	79.82 ^bc^ ± 0.57	4.62 ^a^ ± 0.57	15.57 ^cd^ ± 0.14
OB20	43.58 ^ab^ ± 0.22	5.00 ^b^ ± 0.85	51.43 ^c^ ± 0.37	80.31 ^bc^ ± 0.87	8.98 ^abc^ ± 0.17	10.71 ^b^ ± 0.70

Values are presented as mean ± standard deviation (*n* = 3). Superscripts with different alphabets in the same column show significance (*p <* 0.05) using Duncan’s multiple range test. RAG—rapidly available glucose, SAG—slowly available glucose, UG—unavailable glucose. OB—oat bran, WB—wheat bran. Control denotes fried dough/batter with 0 g bran, while 1–20 represents concentration (g) of bran in product formulation.

**Table 2:** The estimated glycaemic index of *magwinya* samples.

*Magwinya* samples	Wheat bran *magwinya*	
	Fried dough	Fried batter
Control	58.11 ^f^ ± 0.55	80.62 ^f^ ± 0.07
Wheat bran 1	58.39 ^ef^ ± 0.13	65.93 ^a^ ± 0.19
Wheat bran 5	57.97 ^cde^ ± 0.21	66.95 ^b^ ± 0.18
Wheat bran 8	56.46 ^a^ ± 0.05	68.21 ^e^ ± 0.53
Wheat bran 10	57.43 ^bc^ ± 0.11	67.59 ^cd^ ± 0.07
Wheat bran 15	57.11 ^b^ ± 0.65	67.22 ^bc^ ± 0.04
Wheat bran 20	57.62 ^bcd^ ± 0.15	67.84 ^de^ ± 0.33
	**Oat bran *magwinya***	
	**Fried dough**	**Fried batter**
Control	58.11 ^b^ ± 0.55	80.62 ^f^ ± 0.07
Oat bran 1	56.96 ^a^ ± 0.57	80.03 ^ef^ ± 0.42
Oat bran 5	56.34 ^a^ ± 0.66	78.36 ^cd^ ± 0.42
Oat bran 8	56.64 ^a^ ± 0.52	73.57 ^a^ ± 0.10
Oat bran 10	56.65 ^a^ ± 0.47	74.69 ^b^ ± 0.27
Oat bran 15	56.98 ^a^ ± 0.05	77.58 ^c^ ± 0.72
Oat bran 20	57.27 ^ab^ ± 0.32	79.13 ^de^ ± 0.29

Results are presented as mean ± standard deviation (*n* = 3). Mean values with different superscripts in the same column for each type of product (batter/dough) show significance (*p <* 0.05) using Duncan’s multiple range test. RDS—rapidly digestible starch, SDS—slowly digestible starch, RS—resistant starch, TS—total starch, K—constant (dimensionless), HI—hydrolysis index, OB—oat bran. Control denotes fried dough/batter with 0 g bran, while 1–20 represents concentration (g) of bran in product formulation.

**Table 3:** Correlation coefficients of independent variables, glucose, and estimated glycaemic index of wheat and oat bran *magwinya*.

Dependent Variables	Initial Moisture Content	Bran Concentration	RAG	SAG	UG	eGI
Wheat bran *magwinya*						
RAG	0.931 **	−0.310 *				
SAG	0.596 **	0.488 **	0.313 *			
UG	−0.985 **	0.118	−0.957 **	−0.574 **		
eGI	0.865 **	−0.211	0.916 **	0.338 *	−0.892 **	
Fat	0.561 **	−0.087	0.542 **	0.403 **	−0.590 **	0.495 **
Oat bran *magwinya*						
RAG	0.942 **	−0.195				
SAG	0.557 **	0.095	0.397 **			
UG	−0.963 **	0.155	−0.981 **	−0.565 **		
eGI	0.985 **	−0.052	0.962 **	0.518 **	−0.973 **	
Fat	0.636 **	−0.361 *	0.702 **	0.148	−0.661 **	0.655 **

RAG, SAG and UG represent rapidly available, slowly available and unavailable glucose; eGI—estimated glycaemic index; * correlation significance at the 0.05 level, ** correlation significance at the 0.01 level.
